# Clear cell carcinomas of the ovary: a mono-institutional study of 73 cases in China with an analysis of the prognostic significance of clinicopathological parameters and IMP3 expression

**DOI:** 10.1186/s13000-016-0467-5

**Published:** 2016-02-02

**Authors:** Rui Bi, Xuxia Shen, Weiwei Zhang, Yufan Cheng, Zheng Feng, Xu Cai, Wentao Yang

**Affiliations:** Department of Pathology, Fudan University Shanghai Cancer Center, Shanghai, 200032 China; Department of Oncology, Shanghai Medical College, Fudan University, Shanghai, 200032 China; Psycho-Oncology Research & Training (CePORT), School of Public Health, The University of Hong Kong, Pok Fu Lam, Hong Kong; Department of Gynecologic Oncology, Fudan University Shanghai Cancer Center, Shanghai, 200032 China

**Keywords:** Clear cell carcinoma, Prognosis, Stage, Endometriosis, IMP3

## Abstract

**Background:**

Ovarian clear cell carcinoma (CCC) is an uncommon subtype of ovarian epithelial tumor. The prognostic significance of its clinicopathological parameters is discordant, with the exception of stage as the adverse prognostic factor. The present study aimed to evaluate the prognostic significance of its clinicopathological characteristics and the expression of IMP3 (Insulin-like growth factor-II mRNA-binding protein 3, IMP3 or IGF2BP3) in Chinese patients with primary pure CCC.

**Methods:**

We collected clinicopathological data from 73 cases with a minimum of 5 years of follow-up and evaluated the expression of IMP3 by immunohistochemistry.

**Results:**

In total, 49.3 % of the patients were in stage I. Advanced stages were closely related to poor prognosis of disease-free survival (DFS) and overall survival (OS) (*P* < 0.005). Patients with CCC coexisting with endometriosis tended to be younger and to have unilateral involvement but did not exhibit differences in prognosis compared with patients with CCC without endometriosis. Other histological features such as growth pattern, mitosis, and necrosis did not have prognostic significance. IMP3 was positive in 63 % of patients (46 of 73 cases); Thus, positive expression of IMP3 is an adverse prognostic marker in terms of OS (*P* = 0.012), even in stage I patients (*P* = 0.038).

**Conclusions:**

The present study demonstrates that IMP3 expression is a prognostic marker, with the exception of stage. IMP3 represents a biomarker of unfavorable prognosis even in stage I patients.

## Background

The prevalence of ovarian clear cell carcinoma (CCC), which has significant geographic differences, accounts for 5–25 % of cases in various countries [1, 2]. Most of CCC in stage I [3] associated with endometriosis [4] has a poor response to platinum-based regimens, which results in an unfavorable prognosis, particularly for advanced stages [3, 5, 6]. The various morphologies of CCC result in misclassification, which further affects the assessment of morbidity and prognostic factors. A grading system based on CCC growth pattern correlates with survival in ovarian CCC [7]. However, this relationship has not been further confirmed, and the impact of the histopathological characteristics on prognosis has been disputed.

Insulin-like growth factor-II mRNA-binding protein 3 (IMP3 or IGF2BP3), a member of the IMP family, is highly expressed in embryo. This protein is involved in the binding, transport and stability of the embryonic isoforms of the *IGF-II* gene. IMP3 is considered as an oncofetal protein and is related to tumor metastasis. This protein is often found to be overexpressed in many types of tumors, such as gastric cancer, colorectal cancer, liver cancer, and pancreatic cancer, where it acts as an independent prognostic factor [8–11]. However, few studies have examined the role of IMP3 in gynecologic tumors, such as in ovarian CCC, endometrial CCC and cervical squamous carcinomas [12–14]. IMP3 expression in ovarian CCC was reported in a previous study; however, subsequent studies with small sample size have not confirmed the same results [12, 15]. Therefore, the significance of IMP3 expression in ovarian cancer, particularly in CCC, requires further analysis.

Few studies of primary pure ovarian CCC have been examined in Chinese patients and the lack of effective prognostic indicators in addition to staging. Therefore, reviewing and understanding the characteristics of CCC and assessing IMP3 expression as a prognostic parameter are of great importance.

## Methods

### Patients and clinicopathological characteristics

The present study was approved by the Ethical Review Committee of Fudan University Shanghai Cancer Center. Data were collected from 73 patients with a primary diagnosis of pure ovarian CCC who underwent their first operation at Fudan Cancer Center between 1999 and 2009 and who had a minimum of 5 years of follow-up. Hematoxylin-eosin (HE)-stained sections were reviewed by three gynecological pathologists. The patients underwent total abdominal hysterectomy, bilateral salpingo-oophorectomy, omentectomy, and appendectomy simultaneously or asynchronously. The patients were then treated with platinum-based postoperative chemotherapy. Staging was determined according to the 2014 FIGO new ovarian, tubal, peritoneal tumor staging guidelines [16].

Histological morphology was estimated approximately according to the summary of DeLair et al. [17] for the determination of tumor growth pattern (tubulocystic, solid, or papillary), cell type (clear cell, oxyphil cell, hobnail cell or columnar cell), mitotic index (10/HPF), stroma (hyalinized, myxoid, fibroblastic), and necrosis. We also evaluated the presence of endometriosis (ovarian and/or extra-ovarian) in the specimens.

### Immunohistochemistry

IMP3 expression was evaluated by immunohistochemistry using the EnVision method [15]. Briefly, sections (4 μm) were deparaffinized and treated with 0.3 % hydrogen peroxide, boiled in citrate buffer (pH 6.0) for 15 min, and cooled at room temperature. Sections were incubated with a primary IMP3 antibody (Clone 69.1, dilution 1:200, Dako Glostrup, Denmark) at 4 °C overnight, rinsed in PBS, and then incubated for 40 min with biotinylated secondary antibody. Sections were stained in DAB (Dako, Glostrup, Denmark), rinsed three times in PBS, and then counterstained with hematoxylin. Incubation in PBS buffer in lieu of primary antibody was used as a negative control, and IMP3 expression in the germinal center of the lymph node served as a positive control. We defined positive IMP3 staining as convincing cytoplasmic expression in more than 10 % of tumor cells [18]. The positive staining intensity was recorded as weak, moderate, or strong.

### Follow-up

The patients were followed up until March 31, 2014. Overall survival (OS) was defined as the time from operation to either death or the last follow-up. Disease-free survival (DFS) was defined as the interval from operation to disease recurrence or the last follow-up. We defined disease recurrence as a consistent elevation of CA125, or observation of tumor by clincial examination including physical examination and imaging studies [19].

### Statistical analyses

The chi-square test and Fisher’s exact test were used to evaluate the association between IMP3 expression and multiple clinicopathological parameters. Survival analysis was determined using Kaplan–Meier univariate analysis. Differences in survival curves were compared with the log-rank test. *P* values < 0.05 were considered significant. For statistical analyses, SPSS software version 19.0 (IBM SPSS Statistics 19) was used.

## Results

### Clinicopathological characteristics

The ages of the 73 patients ranged from 19 to 80 years old (mean, 53.63 years; median, 54 years). Macroscopically, the tumor size ranged from 2 to 27 cm (mean, 10.45 cm). Unilateral tumors were the most frequent, occurring in 58 cases (79.5 %), while bilateral tumors occurred in 15 cases (20.5 %). The serum level of CA125 was above normal in the 56 preoperative cases (47/56, 83.9 %). Ascites were present in 48.5 % of patients (33/68) and absent in 51.5 % (35 cases) of patients in the recorded data. The percentages of cases at each FIGO stage were as follows: stage I, 49.3 % (36 cases); stage II, 15.1 % (11 cases); stage III, 24.7 % (18 cases) and stage IV, 11 % (8 cases). Follow-up information was obtained from 69 patients. The OS of patients ranged from 0.7 to 173.8 months (mean, 61.2 m; median, 59 m), and the DFS ranged from 0.7 to 173.8 months (mean, 53.2 m; median, 38.2 m). The 5-year survival rate varied among stages as follows: stage I (26/33, 78.8 % of cases), stage II (5/11, 45.5 % of cases), stage III (5/18, 27.8 % of cases), and stage IV (0/6, 0 % of cases) (*P* < 0.001).

**Fig. 1:**
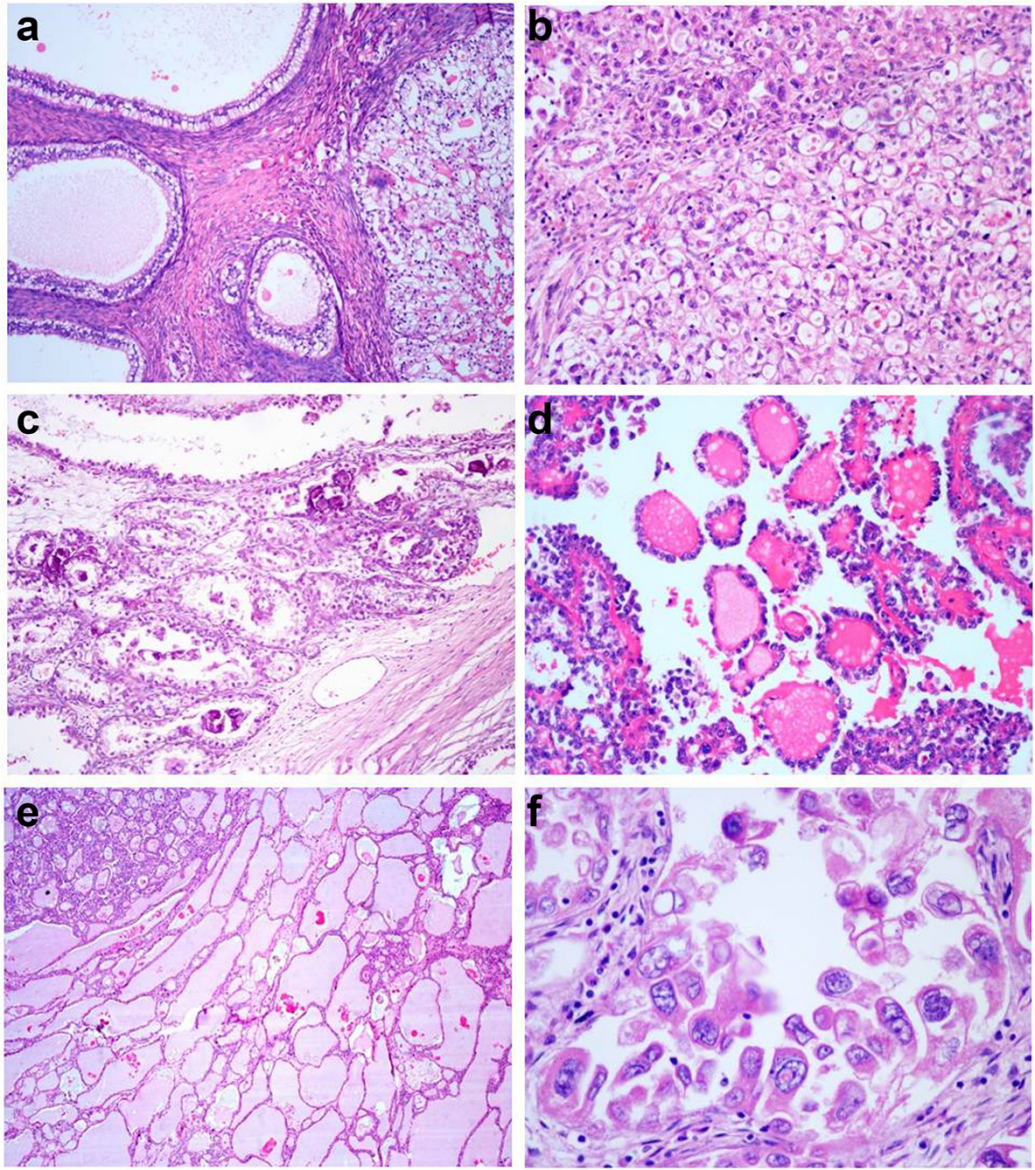
**a** A solid nest is on the right, and the cystic morphology with columnar cells resembles an endometrial gland. **b** Targetoid bodies are signet ring-like cells contained with eosinophil/basophil secretions. Classic hobnail cells can be observed in the top left corner. **c** Scattered psammoma bodies. **d** Open tumor rings consist of a ring of tumor cells without a central fibrovascular stroma (A-D; original magnification, ×200). **e** Extremely expansive tubulocystic morphology with mucinous secretions similar to a thyroid follicle (original magnification, ×40). **f** Nuclear pseudoinclusions were observed with severe cell atypia, but mitotic figures were absent (original magnification, ×400)

Histopathological features were summarized in Table 1. Basically, ovarian clear cell carcinomas showed a variable admixture of papillary, tubulocystic and solid growth pattern. A variable papillary component was present in 82.2 % of cases (60 of 73). Pure papillary pattern, tubulocystic pattern and solid pattern were observed in 34.2 % (25/73), 6.8 % (5/73) and 2.7 % (2/73) cases respectively. Mixed papillary and tubulocystic pattern were present in 27.4 % (20/73) cases. Mixed tubulocystic and solid pattern, mixed papillary and solid were observed in 8.2 % (6 /73) and 8.2 % (6/73) respectively. A mixed pattern of papillary, tubulocystic and solid were present in 12.3 % (9/73) cases. Ovarian clear cell carcinomas were composed by variable proportions of clear cells, oxyphilic cells, hobnail cells and colunmar cells. Hobnail cells were present in 56.2 % (41/73) of all cases. A mixture of clear cells and oxyphilic cells was present in 78.1 % (57/73) cases, Pure clear cell type was observed in 15.1 % (11/73) cases. 6.8 % (5/73) cases composed of pure oxyphilic cell. Rare cases presented with columnar cells with subnuclear or supranuclear vacuoles (Fig. 1a). The mean mitotic index was 4.6/10 HPFs. Mitotic figures were 0–9/10 high-power fields (HPFs) in 65 cases (89 %). The remaining cases had more than 10/10HPF, with the highest of 17/10 HPFs. Diversity was observed among the stromal changes, including fibrous, hyaline and myxoid stroma. Pure hyaline or myxoid stroma were observed in 4 cases (5.5 %) and 1 case (1.4 %) respectively. Focal or patchy necrosis was observed in 39.7 % (29/73) of the cases. Targetoid bodies (signet ring-like cells with eosinophil/basophil secretions, shown in Fig. 1b), psammoma bodies (Fig. 1c), and open tumor cell rings (rings of tumor cells without central stroma, Fig. 1d) were presented in some cases. Tubulocysts filled with mucinous secretions were observed in eight cases, one of which with severe expansile tubulocysts resembling thyroid follicles (Fig. 1e). Scattered nuclear pseudoinclusions were noted in 6 cases (Fig. 1f). Endometriosis was observed in the specimen in 23.3 % (17 of 73) of the cases.

**Table 1:** Morphological characteristics in 73 cases of ovarian clear cell carcinoma

Morphology	Cases (%)
Growth pattern	
Tubulocystic	5 (6.8)
Solid	2 (2.7)
Tubulocystic/Solid	6 (8.2)
Papillary	25 (34.2)
Papillary/Tubulocystic	20 (27.4)
Papillary/Solid	6 (8.2)
Tubulocystic/Solid/Papillary	9 (12.3)
at least focal papillary componen	*60 (82.2)*
Cell type	
Clear cell	11 (15.1)
Oxyphil cell	5 (6.8)
Mixed with Clear and oxyphil cell	57 (78.1)
Hobnailing	41 (56.2)
Columnar cell	2 (2.7)
Mitotic figures (/10 HPF)	
0–9	65 (89.0)
10–19	8 (11.0)
Stroma	
Hyalinized only	4 (5.5)
Myxoid only	1 (1.4)
Fibroblastic only	23 (31.5)
Fibroblastic/Hyalinized	20 (27.4)
Fibroblastic/Myxoid	4 (5.5)
Hyalinized/Myxoid	1 (1.4)
Fibroblastic/Hyalinized/Myxoid	20 (27.4)
Other miscellaneous characteristics	
Focal or patchy necrosis	29 (39.7)
Targetoid bodies	3 (4.1)
Psammoma bodies	8 (11.0)
Mucin secretion	8 (11.0)
Open tumor rings	6 (8.2)
Nuclear pseudoinclusions	6 (8.2)

### Clinical parameters and survival (Table 2)

**Table 2:** The association of the clinical features and the expression of IMP3 with the prognosis of 73 patients with ovarian clear cell carcinoma

		DFS	OS	IMP3 expression			Endometriosis		
		*P*	*P*	Positive	Negative	*P*	Present	Absent	*P*
Age						0.097			0.071
	≥60y	0.084	0.073	17	5		2	19	
	<60y			29	22		15	36	
Tumor size						0.222			0.415
	≥15 cm	0.337	0.285	8	8		5	11	
	<15 cm			38	19		12	44	
Lateral						0.786			0.038
	Unilateral	/	/	37	21		40	17	
	Bilateral			9	6		15	0	
CA125						0.115			0.375
	Above normal	0.201	0.935	29	18		10	34	
	Normal			8	1		3	5	
Ascites						0.347			0.521
	Present	0.055	0.271	19	14		9	7	
	Absent			24	11		24	27	
Endometriosis						0.352	/	/	/
	Present	0.534	0.424	9	8				
	Absent			36	19				
Necrosis						0.260			0.296
	Present	0.377	0.499	16	13		5	24	
	Absent			30	14		12	31	
Papillary component						0.188			0.631
	Present	0.123	0.180	37	18		12	42	
	Absent			9	9		5	13	
Mitotic figures (/10 HPF)						0.488			0.660
	<10	0.712	0.947	40	24		12	44	
	≥10			6	2		1	6	
Stage						0.074			0.038
	I	<0.001	<0.001	19	17		12	23	
	II–IV			27	10		5	32	
DFS				44	18	0.175			
OS				44	18	0.012			

Follow-up information was obtained for 69 patients, including 33 in stage I, 11 in stage II, 18 in stage III, and 7 in stage IV. The mean OS of each stage was as follows: I, 85.3 months; II, 57.15 months; III, 31.5 months; and IV, 30.1 months. Statistically significant differences in the mean OS were observed (*P* < 0.001). The mean DFS of each stage was as follows: I, 79.9 months; II, 45.4 months; III, 24 months; and IV, 14 months. The differences in the mean DFS were also significantly different (*P* < 0.001). The survival of patients with stage I disease resulted in a remarkably favorable prognosis compared with that of patients in stages II–IV; we then analyzed the data from the two groups in stages I and II–IV. The comparison of DFS and OS between the patients in stages Ia/Ib and Ic showed no statistically significant differences (*P* = 0.440 and *P* = 0.875, respectively).

Moreover, patients with ovarian CCC coexisting with endometriosis were prone to develop unilateral tumors (*P* = 0.038), with a trend of occurrence in relatively younger patients (15 patients of < 60-year-old group vs. 2 patients of ≥ 60-year-old group, *P* = 0.062). Other clinical parameters, such as age, tumor size, preoperative CA125 level, ascites, and endometriosis, exhibited no significant difference in their association with DFS and OS between groups (Table 2).

### IMP3 immunohistochemical staining and survival

**Fig. 2:**
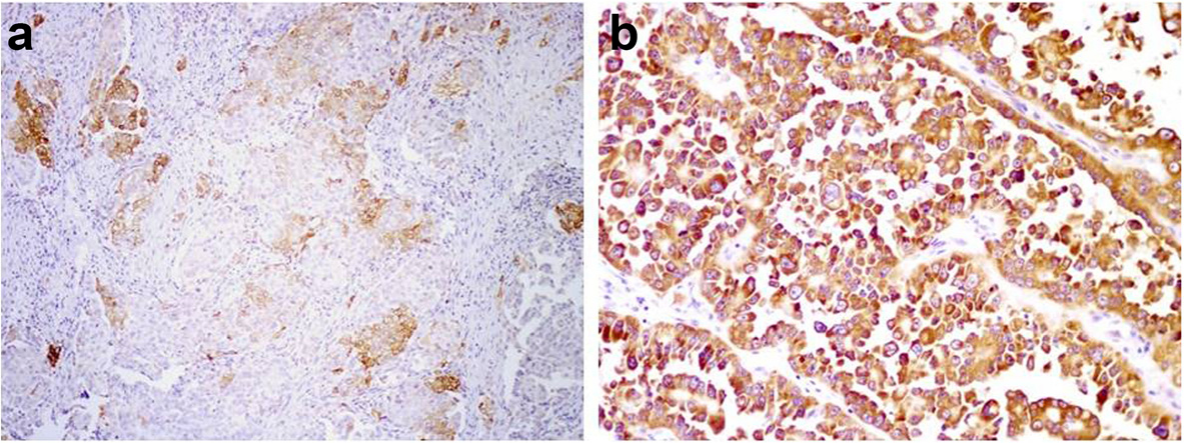
**a** Focal and moderately positive staining for IMP3 expression. **b** Diffuse and strong IMP3-positive staining (original magnification, ×200)

Forty-six of the 73 cases (63 %) showed positive IMP3 expression (Fig. 2a and 2b), and the remaining 27 cases (37 %) were IMP3 negative.

**Fig. 3:**
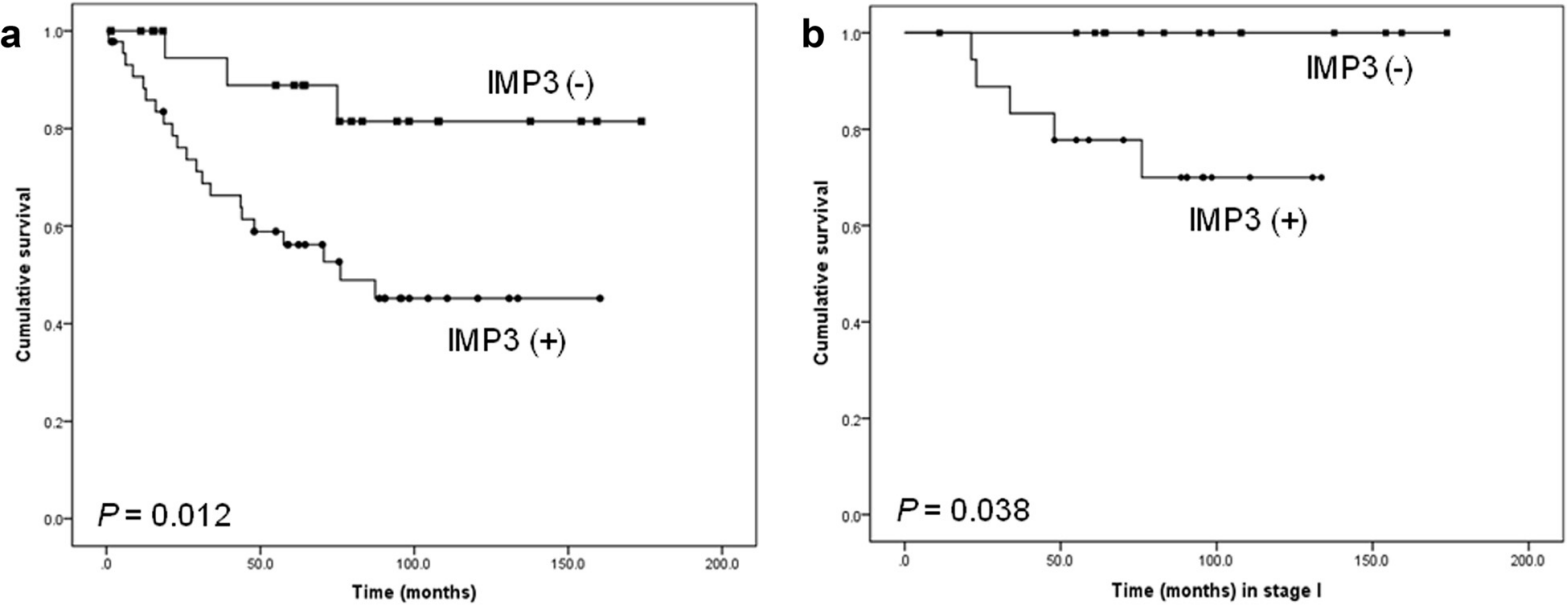
**a** The OS of the patients with positive IMP3 expression was worse than that of the negative cases among 73 cases. **b** The same trend was observed in 36 cases with stage I disease

The rate of IMP3 expression was significantly higher in patients with an advanced stage (II–IV), with 73.0 % positive cases (27 of 37), compared with the 52.8 % of cases observed in stage I (19 of 36) (*P* = 0.046). The OS was significantly shorter in IMP3-positive patients than in those negative cases (55.8 months vs. 71.3 months, *P* = 0.012, Fig. 3a), but no significant difference was observed for DFS (*P* = 0.175). Upon further stratification analysis of IMP3 expression in patients with stage I, the IMP3-positive cases (23–133.6 months, mean 76.0 months) had a shorter OS than did the IMP3-negative cases (11.2–173 months, mean 96.5 months) (*P* = 0.038, Fig. 3b). None of the 17 IMP3-negative cases of stage I died. However, 4 of 19 IMP3-positive cases died from ovarian CCC metastasis, and their OS ranged from 23.0 to 76 months (mean 40.4 months). Nevertheless, the DFS did not differ significantly between IMP3-positive and IMP3-negative cases (*P* = 0.557). However, no statistically significant differences in OS and DFS were observed in patients with stage II-IV CCC (*P* = 0.892 and *P* = 0.840, respectively).

## Discussion

Compared with common serous carcinomas, ovarian CCC has distinct features such as poor prognosis and platinum drug resistance. A relatively high percentage of CCC cases are in stage I (31–-60 %) [20–23], which is significantly higher than the percentage of patients who have serous carcinoma (16.6–20 %) [3, 24]. Stage I cases accounted for approximately 45.8–62.8 % of Chinese CCC patients [25, 26]. The percentage of pure CCC patients in stage I in the present study was 49.3 % (36 of 73), which is similar to the percentages that have been reported in other populations.

According to a report by Higasi et al., the five-year DFS and OS rates of each stage of ovarian CCC were 84 % and 88 % (stage I), 57 % and 70 % (stage II), 25 % and 33 % (stage III), and 0 % and 0 % (stage IV), respectively [27]. The five-year OS rates of the patients in our group were similar to those reported in other studies of non-Chinese populations: 78.8 % (stage I), 45.5 % (stage II), 27.8 % (stage III), and 0 % (stage IV). According to a previous report of a Chinese population, the five-year OS rate of patients with stage I was 66.8 % [25]; the survival rates in the current study were higher than those in previous reports. A study by Hoskins et al. indicated that the prognosis of patients with CCC in stage Ia/Ib was more favorable than that of patients in stage Ic [28] and that the prognosis of patients with stage Ic was closer to that of patient with stage II [29]. However, OS and DFS rates did not significantly differ between stages Ia/Ib and Ic in this group; this result was comparable to the report by Hoskins et al.

Ovarian CCC is closely related to endometriosis, which was found in 23.3 % of our cohort. This value is similar to the frequency of 26.8 % reported in another Chinese population [26] but lower than that reported in other countries (approximately 45 %) [30, 31]. Scarfone et al. reported that patients with CCC arising from endometriosis are generally younger than those with general CCC [32]. Our results indicate that CCC cases coexisting with endometriosis also had a trend of occurrence among relatively younger patients and were often unilateral, although few cases arose directly from endometriosis. The presence or absence of endometriosis was not related to prognosis in any case in our study or in a previously reported study [32]; however, some studies have observed a better prognosis in CCC-associated endometriosis [33, 34].

Ascites were often observed during operation and were a trend among the patients with an unfavorable prognosis compared to patients without ascites with respect to DFS (*P* = 0.055) but not OS (*P* = 0.271).

The classical morphology of ovarian CCC cases is tubulocystic, solid and papillary or with an admixture of differnt growth pattern [17]. In the present study, 72 % of the cases contained papillary structures. A mixture of clear cells and oxyphilic cells was the most common cell type. The tumor stroma was often observed to be a mixture of hyalinized, myxoid, or fibroblastic types, whereas pure hyalinization or pure myxoid stroma was uncommon. In our cases, the average mitotic index was 4.6/10 HPFs, with most cases being lower than 10/10 HPFs (89 % of the group), which is quite consistent with previous reports [31]. Furthermore, we did not observe any statistically significant differences in the relationship between morphology and prognosis.

The importance of IMP3 expression in the female reproductive system is controversial. One study suggested that IMP3 was a prognostic marker in metastatic ovarian cancer [35], Kobel et al. suggested that IMP3 expression was an independent indicator of a poor ovarian CCC prognosis [12], and Noske et al. suggested that IMP3 expression was a marker of a good prognosis in ovarian cancer (including serous carcinoma and non-serous carcinoma) [15]. No further reports regarding IMP3 as a prognostic biomarker have been published. The present study demonstrated that IMP3 expression is closely related to an unfavorable prognosis and that it may be used as a potential indicator to estimate poor prognosis. The results of our study are similar to those of Kobel et al. In endometrial clear cell carcinoma, IMP3 was reported to be closely correlated with poor prognosis and relapse free survival (RFS) [13].

Furthermore, we analyzed the follow-up data of 33 patients with stage I CCC and found that IMP3 positivity was associated with a shorter OS (76.0 months vs. 96.5 months, *P* = 0.038). None of the 17 IMP3-negative cases of stage I died, but 4 of 19 IMP3-positive cases died from ovarian CCC metastasis. The results for the 5-year survival rate in our series show the same trend reported by Kobel et al. Most ovarian CCC patients received adjuvant chemotherapy after surgery. But for those stage I patients, the benefit from chemotherapy was not sufficient [36, 37]. However, the patients may have, to endure severe side effects of chemotherapy. IMP3, as a potential stratification prognostic factor in stage I cases, may be used to stratify patients whom may benefit from adjuvant therapy. However, IMP3+ tumors in stage I patients still have a wide range of survival (23–133.6 months) in our study. Thus, further research are needed to verify the prognostic significance of IMP3 in ovarian CCC. For example, studies are needed to demonstrate whether IMP3 staining correlates well with molecular tests for IMP3 expression, and furthermore, that IMP3 expression by molecular test correlates with prognosis.

It had been reported that knockdown of IMP3 in cervical cancer, breast cancer and melanoma cell lines could reduce cell migration and invasiveness [38–40]. In renal cell carcinoma, IMP3 can also promote cell migration and invasion by activation of NF-κB pathway [41]. As recently reported, knockdown of IMP3 decreased cell proliferation, migration, and invasion, and increased the sensitivity to platinum in ovarian cancer through increased expression of hCTR1 [42]. We presume that the overexpression of IMP3 can increase the resistance to platinum, which may lead to poor prognosis in ovarian CCC, but further study is required.

## Conclusion

In conclusion, we demonstrated that tumor stage and IMP3 expression are prognostic indicator in ovarian CCC. IMP3 overexpression is a potential marker of poor prognosis for ovarian CCC, even in stage I. More studies are required to further clarify the mechanism of IMP3 expression and its significance in ovarian CCC.
